# What Lies INSIDE: Chemometric Insights on the Penetration Depth of Near-Infrared Radiation in Spectral Imaging Configurations

**DOI:** 10.1021/acs.analchem.6c00848

**Published:** 2026-06-25

**Authors:** Sara Gariglio, Cristina Malegori, Paolo Oliveri, Monica Casale, Carolina Scagliarini, Alberto Mazzoleni, Eugenio Alladio, Zelan Li, Emilio Catelli, Giorgia Sciutto, Aoife Gowen, Sergey Kucheryavskiy

**Affiliations:** a Department of Pharmacy (DIFAR), University of Genova, Viale Cembrano 4, Genova 16148, Italy; b Department of Chemistry and Industrial Chemistry (DCCI), University of Genova, Via Dodecaneso 31, Genova 16146, Italy; c Department of Chemistry, University of Torino, Via Pietro Giuria 7, Torino 10125, Italy; d Department of Chemistry “Giacomo Ciamician”, 9296 University of Bologna, Via Guaccimanni 42, Ravenna 48121, Italy; e School of Biosystems and Engineering, 8797 University College Dublin, Belfield, Dublin 4 D04 V1W8, Ireland; f Department of Chemistry and Bioscience, Aalborg University, Niels Bohrs Vej 8, Esbjerg 6700, Denmark

## Abstract

Understanding how deeply near-infrared (NIR) radiation penetrates into matter is essential for interpreting (hyper)­spectral imaging (SI) data, yet comprehensive assessments of penetration depth remain limited. Although NIR-SI is traditionally considered a surface analytical technique, subsurface contributions may affect spectral profiles, challenging this assumption. This study presents a systematic and quantitative investigation of NIR penetration in a controlled SI setup using multilayer polymeric samples manufactured by 3D printing. Layered cubes and cylinders composed of polylactic acid (PLA) and polyethylene terephthalate glycol (PETG) were analyzed using two independent short-wave NIR-SI systems. Penetration behavior was evaluated through an integrated chemometric workflow combining exploratory analysis (principal component analysis, PCA), unmixing (classical least squares, CLS), supervised linear (partial least squares, PLS) and nonlinear (convolutional neural network, CNN) regression methods. The results demonstrated that NIR-SI can retrieve chemical information from subsurface layers to depths of ∼1 cm. Penetration depth was strongly influenced by material composition, internal structure, and illumination conditions. PCA and CLS revealed nonlinear attenuation and scattering phenomena, while regression models successfully retrieved subsurface information across the full sample height. CNN consistently outperformed PLS, highlighting the importance of nonlinear modeling approaches. The robustness of the proposed strategy arises from the use of designed ad hoc samples, two independent instruments, and multiple complementary chemometric methods converging toward consistent results. Overall, this work challenges the conventional surface-limited view of NIR-SI and provides a robust framework for investigating NIR penetration, supporting the development of depth-resolved and potentially tomographic NIR-SI approaches.

## Graphical abstract

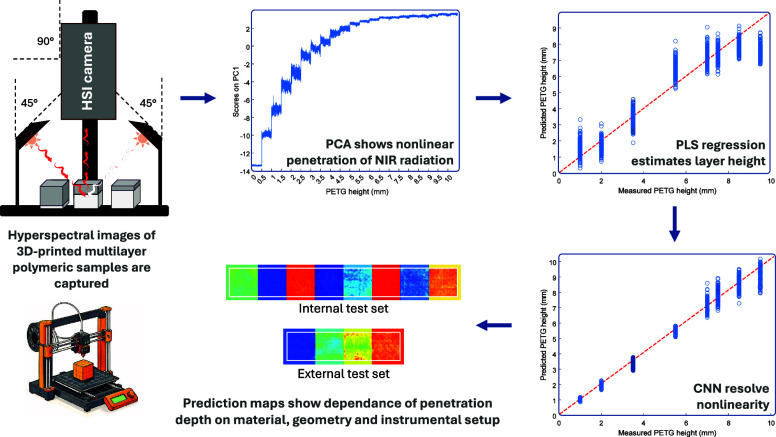

The fundamental objective of imaging techniques is to capture and visualize characteristics of a sample spatially distributed over a surface or a scene. In conventional imaging, these characteristics are mainly related to color components (e.g., hue, saturation, lightness, etc.),^1^ while in the context of spectroscopic imaging, this objective is broadened to encompass the simultaneous acquisition of spatial and spectral information, thereby generating a spectral profile at each pixel. Instruments employed for this purpose are based on most of the primary spectroscopic techniques of analytical relevance, such as ultraviolet–visible, fluorescence, near-infrared (NIR), mid-infrared, Raman and X-ray fluorescence.^2^ Collectively, these methodologies fall under the broad classification of hyperspectral imaging (HSI), or, as stated by Polder & Gowen,^3^ more appropriately described by the umbrella term *spectral imaging* (SI), which avoids ambiguous distinctions based solely on the number of spectral bands and better reflects the underlying concept of spatially resolved spectroscopy. These techniques are typically nondestructive, contactless analytical methods and are particularly suitable for the study of delicate or high-value specimens, as well as for automation in industrial contexts.

Conventionally, SI entails the acquisition of two spatial dimensions (x, y) in conjunction with a spectral dimension (λ). However, while the related data sets are considered to primarily represent surface information, they may inherently contain signals related to subsurface material composition, as radiation at specific wavelengths can penetrate below the surface. Within the spectral regions employed in analytical spectroscopy, NIR radiation has been shown to exhibit penetration capabilities.^4^

NIR spectroscopy is a rapid, noninvasive and nondestructive analytical technique with broad applicability across diverse domains, including medical diagnostics, food quality assessment, forensic analysis, and cultural heritage investigations.^5^ It operates within the electromagnetic spectrum range of 780 to 2500 nm (12,820 to 4000 cm^–1^) and is based on the absorption of radiation by vibrational overtones and combination bands of fundamental molecular vibrations. The relatively low energy of NIR photons means they seldom correspond to strong electronic transitions, leading to weaker absorption^5^ and consequently deeper penetration of radiation through materials. The effective penetration depth of NIR radiation is, however, influenced by multiple factors beyond chemical composition, including scattering, sample geometry, radiation intensity and incidence angle, and the specific wavelengths concerned.^4,6,7^ A comprehensive understanding of these parameters is essential for optimizing the application of NIR spectroscopy across various scientific and technological fields.

Until now, some studies have explored the penetration depth of NIR radiation in biomedical, pharmaceutical, cultural heritage, and food science applications. For instance, in the field of brain imaging research, optical penetration in the Vis-NIR range (488–1060 nm) has been shown to be greater in neonates than in adults due to age-related differences in brain tissue composition, particularly myelination.^8^ In adult transcranial NIR spectroscopy, depths of approximately 2–3 cm within the head are reached, with most of the detected signal originating from superficial tissues such as scalp and skull.^9^ On the other hand, when studying noninvasive glucose monitoring, effective penetration was limited to a few millimeters, reportedly due to high water content in skin tissues.^10^ Cartilage analysis pointed out a wavelength-dependent behavior of penetration, which was up to 5 mm at 1110–1430 nm and decreased at longer wavelengths.^11^ Wavelength dependence of penetration was also confirmed in pharmaceutical studies: among others, Clarke et al. found penetration depths ranging from ∼0.1 mm at 2500 nm to ∼0.8 mm at 1100 nm in cellulose-based materials,^12^ while Yang et al. observed penetration up to 2 mm in tablets,^13^ which was influenced by formulation and wavelength selection. The topic has also been investigated in food analysis,^14,15^ where NIR radiation penetration into fruit peel was shown to be wavelength-dependent, demonstrating good penetration across different types of fruits. NIR radiation was, for instance, shown to penetrate 9–15 mm in watermelon, depending on sample configuration,^16,17^ and up to 1.8 mm in highly scattering powdered products like wheat flour.^18^ Moisture content emerged as a key factor, reducing penetration in high-moisture samples such as raw potato (1 mm) versus ham (2.4 mm).^19,20^ In cultural heritage analysis, Longoni et al. indirectly estimated penetration depths of 60–100 μm using FT-NIR (7500–4000 cm^–1^) in multilayered paintings,^21^ while Cucci et al. and Catelli et al. extended NIR-HSI (750–2500 nm) for stratigraphic imaging, demonstrating enhanced subsurface visualization capabilities.^22,23^

While these studies provide valuable application-specific insights, to date only a limited number have attempted to characterize NIR penetration depth systematically and quantitatively. Among them, Pomerantsev et al.^24,25^ proposed a deconvolution strategy to separate transmittance, scattering, and absorbance contributions, but their work primarily aimed at identifying the spectral signature of subsurface targets rather than elucidating the physical mechanisms of penetration. Moreover, most studies employed point-based NIR spectroscopy rather than hyperspectral imaging, thus neglecting the potential of spatially resolved data for investigating penetration phenomena.

Building on this considerations, the present study, developed under the two-year *INSIDE* project funded by the Italian Ministry of Universities and Research, addresses this critical gap. This ambitious project aims to evolve SI from a surface analytical technique into a depth-resolved, three-dimensional spectral tomography. In this context, the present work provides the first systematic and quantitative investigation of NIR radiation penetration in a controlled SI configuration, using layered polymeric samples with known stratigraphy and composition produced via 3D printing. Light penetration behavior was examined using complementary chemometric strategies, including exploratory (principal component analysis – PCA), unmixing (classical least squares – CLS), and both linear (partial least squares – PLS) and nonlinear (convolutional neural networks – CNN) regression approaches. To verify the consistency of the outcome, analyses were performed in parallel using two different NIR-SI instruments.

## Materials and Methods

### Sample Production

In-house layered samples were printed using an Original Prusa i3MK3S + MMU3 3D printer (Prusa Research a.s., Prague, Czech Republic), coupled with the PrusaSlicer-2.9.2 software for slicing and assembling the samples. All projects were realized in the OpenSCAD 2021.01 environment.

The printing materials (Prusament from Prusa Research a.s., Prague, Czech Republic) were acquired as spools with a filament diameter of 1.75 ± 0.02 mm. Two different polymers were used to produce the samples: polylactic acid (Transparent PLA, 1 kg spool) and polyethylene terephthalate glycol (Transparent PETG Clear, 1.038 kg spool). These two polymers were chosen from the available options because they proved easy to print and presented distinct spectral signatures in the NIR range.

A total of 21 samples was produced in the form of 1 cm cubes. The first cube was made entirely of PLA, then layers were prepared, increasing the height of PETG by 0.5 mm and consequently decreasing the height of PLA by the same amount (or “pace”) for each sample until the last cube, which was made entirely of PETG. Figure 1A shows a schematic representation of the series. The cubes were made as solid samples, i.e., without space within the structure (100% infill density), and subsequent layers were printed with a linear infill pattern, as represented in Figure 1D.

**Figure 1:**
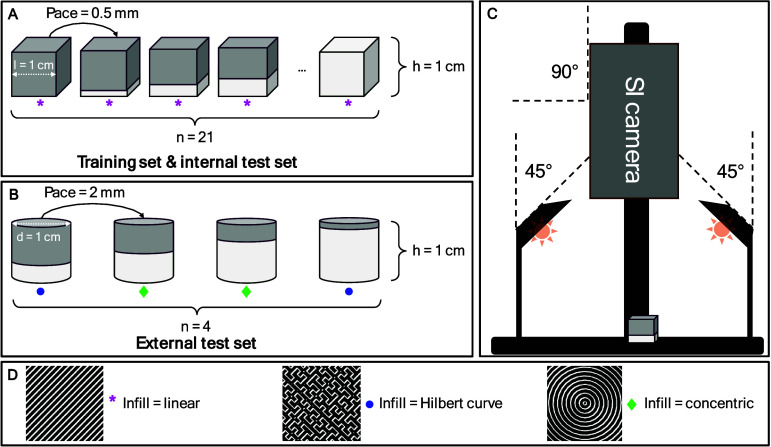
Schematic representation of the samples, their printing pattern and the instrumental configuration. A: Cubic samples, showing the increase of PETG (light gray) of 0.5 mm and consequent decrease of PLA (dark gray) along the series, keeping the total height constant (1 cm); B: Same as A but for cylindrical samples; C: Schematic representation of the SI acquisition setup; D: Detail of the infill patterns.

Furthermore, four additional layered samples were printed in the shape of cylinders with a 1 cm base diameter and a total height of 1 cm, with a pace of 2 mm (Figure 1B). They were printed with a 100% infill density, but the infill patterns differed from those employed in the cubes. Specifically, the cylinders with 2 and 8 mm of PLA employed the Hilbert curve infill pattern, while the cylinders with 4 and 6 mm of PLA used the concentric infill pattern. Both patterns are represented in Figure 1D. This further set was produced with the idea that the printing pattern, affecting the physical properties of the material, might influence how the radiation interacts with it, modifying scattering effects. Therefore, the production of new samples with the same infill density but different shape and printing patterns ensured the presence of a truly independent set of samples to be used for model validation.

### Image Acquisition

NIR-SI images were acquired using two different push-broom short-wave NIR-SI systems, which exhibited comparable configurations (Figure 1C).

In particular, the first instrument was a SWIR3 spectral camera from Specim (Spectral Imaging Ltd., Oulu, Finland), while the second was a SWIR-384 spectral camera from HySpex (NEO HySpex, Oslo, Norway). Both instruments operated within the 960–2500 nm spectral range and were equipped with the same MCT (mercury–cadmium-tellurium) sensor with cooling to 150 K, characterized by 384 spatial pixels and 288 spectral channels (FWHM = 5.5 nm). The first camera mounted an OLES15 lens (focal length = 50 mm), while the second camera mounted a SWIR-320/384 close-up 30 cm lens assembly (focal length = 30 mm), positioned at a distance of 20 and 17.6 cm from the samples, respectively. The samples were illuminated with six tungsten halogen lamps (Osram, Styria, Austria) (12 V, 35 W, 2900 K, 430 Lm) in the first instrument, while the second instrument was equipped with four tungsten halogen lamps (12 V, 150 W, 3100 K, ∼600 Lm), custom-made at HySpex. In both cases, the lamps covered the 400–2500 nm spectral range and were positioned at a 45° angle, at approximately 25 cm from the samples, with half of the lamps situated in front and the other half located behind them.

The image acquisition parameters were set with the goal of maximizing signal intensity while avoiding pixel saturation. The Specim system was therefore configured as follows: exposure time of 6 ms, frame rate of 70 Hz, scanning speed of 28.2 mm/s. Conversely, the HySpex configuration presented exposure time set to 4.8 ms, frame rate to 185 Hz and scanning speed to 728 mm/s. The differences in the parameters are connected to differences in lenses’ field of view (50 mm for Specim, 30 mm for HySpex), with consequent differences in magnification, and to the discrepancies in lamp intensities. With the described conditions, the resulting pixel sizes for the Specim and HySpex spectrometers were approximately 0.4 and 0.2 mm, respectively. The Lumo Scanner V. 2.6 software (Specim, Spectral Imaging Ltd., Oulu, Finland) and HySpex Ground Software v4.9.3.8 (NEO HySpex, Oslo, Norway) were utilized to regulate the two systems.

Prior to the acquisition of sample images, both instruments recorded a white reference image, W (99% reflectance Spectralon) and a dark image, D (closed shutter). The reflectance of each pixel was then computed as delineated in eq 1:

$$R_{\lambda }=\frac{{I_{\lambda }}_{Sample}-{I_{\lambda }}_{D}}{{I_{\lambda }}_{W}-{I_{\lambda }}_{D}}$$ 1

where *R*_*λ*_ is the reflectance value calculated for each wavelength, *I*_*λSample*_ is the intensity at each wavelength of the profile recorded for the sample, *I*_*λD*_ is the intensity at each wavelength of the dark image, and *I*_*λW*_ is the intensity at each wavelength of the white reference image.

For data acquisition, samples were first arranged on the instruments’ moving stage with the PETG surface oriented toward the detector. This configuration will be referred to as PETG-UP in the text. Subsequently, the samples were turned upside down, and the data acquisition was repeated, with the PLA surface facing the detector. This configuration will be referred to as PLA-UP in the text.

For each measurement, the focal point of the instrument was recalibrated to align with the lowermost layer of the samples. However, the depth of field of the two instruments was sufficiently broad to ensure optimal focus on the uppermost portion of the samples as well. Initially, images were acquired for the set comprising the twenty-one cubes. This set of images will be referred to as the “training set” in the text. In the subsequent phase, eight cubes were randomly selected from the training set and resubjected to imaging, with the images being captured in random order. This set of images will be referred to as the “internal test set” in the text. Finally, the set containing the four cylindrical samples was imaged. This set will be referred to as the “external test set” in the text. The polymers’ height in the samples of each set is detailed in Table S1 of the Supporting Information.

This acquisition protocol ensured the production of three independent sets of images with defined roles in the subsequent chemometric treatment. In particular, the training set images will be used in all model development steps. The two test sets of images will be used in model validation. In particular, the internal test set, including the same samples as the training set but in a random order and on a reduced range, serves as a first check for model adequacy, while the external test set, being composed of new samples in terms of printing properties and shape, represents a truly independent set to be used for overfitting evaluation and general performance testing, with the final goal of evaluating the effect of physical properties on NIR radiation penetration depth.

### Data Processing

All data preprocessing and chemometric analyses were carried out using the PLS_Toolbox 9.5 software (Eigenvector Research Inc., Manson, WA, USA) and in-house scripts developed both under the MATLAB environment, version R2024b (MathWorks, Natick, Massachusetts, USA), and under the Python environment, version 3.12.7 (Python Software Foundation, Wilmington, Delaware, USA). All the steps detailed in the following sections were operated in parallel for both instruments and for the two configurations, PETG-UP and PLA-UP.

#### Masking and Segmenting

The first step of image processing consisted of selecting square regions of interest (ROIs) from each sample, with the aim of removing the background while excluding the effect of sample borders, which may exhibit high scattering and present a different printing pattern. In particular, for Specim data 16 × 16 pixels ROIs were extracted from each sample, while for HySpex data 26 × 26 ROIs were extracted for the training and internal test sets, and 22 × 22 pixels ROIs were extracted for the external test set. The obtained ROIs were then concatenated to obtain a separate matrix containing all the selected pixels for each of the sets. Details on the dimensions of each matrix can be found in Table S1 of the Supporting Information.

The data processing pipeline is reported in Figure 2.

**Figure 2:**
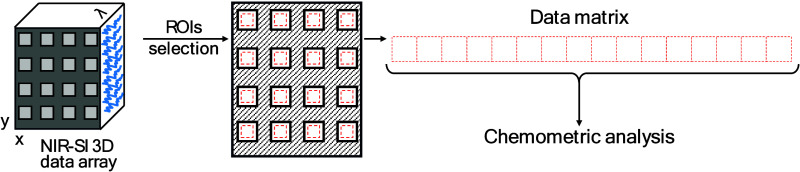
Schematic representation of the data processing pipeline, in which each square in the leftmost image represents a sample at one wavelength.

#### Exploratory Analysis

Initially, pixel spectra from each ROI were averaged to obtain an average signal for each sample. This was done following an object-based approach,^26,27^ to reduce the matrix dimensionality, thus enhancing graph readability and data interpretability. The obtained spectra were plotted in their raw form or preprocessed by means of the standard normal variate (SNV) transform.^28^ The evolution of spectral signatures in relation to polymer height was evaluated.

Subsequently, all pixels from the ROIs were submitted to further data analysis, employing a pixel-based approach.^26,29^ The pixel-based approach was preferred because the samples were produced using a printing technique that ensures uniform polymer height across the sample section. Therefore, each pixel was treated individually, though keeping in mind its belonging to a single physical object. The training set was then subjected to PCA.^30^ The PCA model was developed on either the PETG-UP and PLA-UP configurations together as a single set of data or keeping the two sets separate. In this step, different preprocessing techniques were employed to minimize unwanted scattering effects while enhancing the information regarding polymer height, and the combination best enhancing the information of interest, i.e., describing it in the first principal component with the highest associated variance, was selected for further data processing. Afterward, the scores obtained for each pixel were rescaled between 0 and 1 and encoded with a color scale from blue to red, to reconstruct the sample image, thereby yielding a score map. The score map was instrumental in identifying the origin of variability and in providing insights into the relationship between score values and polymer height. In addition, loading plots were extracted and compared to the object-based spectra.

Afterward, the internal test set and external test set data were projected into the training set’s principal components space to produce score maps. The projection was carried out centering the test set data according to the training set average, thus obtaining centered test set data (**X**_c, test_), then score values for the test sets (**T**_test_) were calculated from the loadings (**P**) as follows:

$$T_{\mathrm{test}}=X_{c,\mathrm{test}}P$$ 2

#### Quantitative Analysis

Next, the preprocessed spectra from all sets underwent classical least squares (CLS)^31,32^ analysis, also known as ordinary least squares (OLS), for spectral linear unmixing. In particular, the CLS technique was employed to extract a predicted height of the two polymers in each sample through use of the pure-component spectra (**S**), represented by the mean spectra of the two single-material cubes.^33,34^ The predicted concentrations (**C**) were calculated as follows:

$$C=XS{\left(S^{T}S\right)}^{-1}$$ 3

where **X** is the matrix containing the spectra.

The height of the polymer facing the detector was then compared with the known polymer height in the sample. The deviation from the true value was calculated using root-mean-square error (RMSE). Then, the height of the top polymer estimated by the model for each pixel was encoded with a color scale from blue to red, and used to reconstruct the image, obtaining a prediction image. In order to facilitate comparison of predicted and expected values, each predicted image was supplemented with a border representing the color of the expected value, i.e., the color that the ROI should have if the prediction had an RMSE of 0. Therefore, the more the inner color visually matches the border color, the more precise the prediction is. An example of this visualization is shown in Figure 3, where some ROIs (1, 2, 3, 6, 8) show good predictive performance, while others (5) show systematically underestimated values, and others (4, 7) show systematically overestimated values.

**Figure 3:**
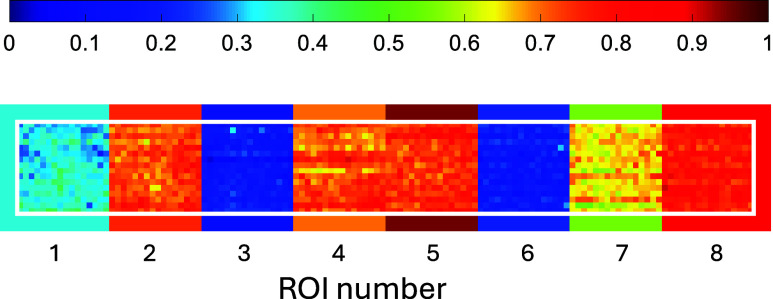
One example of the graphic representation of prediction maps, including a border which represents the color the corresponding ROI should have if its prediction error was null.

Subsequently, two regression models were calibrated to predict the height in millimeters of the polymer facing the detector. In this case, the matrix of predictors, **X**, was represented by the pixel spectra, while the vector of responses, **y**, which served as a reference for the regression calculation, corresponded to the height of the top-polymer layer in millimeters.

The first approach applied was a linear regression method, i.e., PLS.^35,36^ The model was cross-validated using a leave-one-level-out approach,^29,37^ i.e., the cross-validation scheme was designed to remove from each iteration one entire ROI representing a specific Y value and use it for prediction. This strategy circumvents the potential issue of duplicate pixels in the calibration and cross-validation sets, thereby ensuring a more reliable estimate of prediction error (RMSEP) through RMSE in cross-validation (RMSECV). The number of significant latent variables (LVs) was determined based on RMSECV, i.e., choosing the first minimum value of RMSECV minimizing the RMSECV/RMSEC ratio as well. The associated regression vectors and Variables Importance in Projection (VIP) were considered for spectral interpretation.

Conversely, the second regression approach employed was nonlinear, namely CNN.^38,39^ Preprocessing was evaluated again, since it can be in part dealt with by convolutional layers themselves,^40,41^ and therefore signals might benefit from a milder preprocessing. The network structure was optimized to guarantee robustness and repeatability, and it consisted of three convolutional layers followed by three fully connected layers. The connection between subsequent layers was regulated by the ReLU function,^42^ and the Adam optimizer^43^ from the Python Torch library was chosen for backpropagation and weight optimization, with mean squared error (MSE) as the loss function.

Cross validation during model training was carried out on a random subset selected from the training set by means of the DataLoader function from the PyTorch library and a batch size of 200. The number of epochs was set to 70, and the learning rate was gradually adjusted during the training process by means of a scheduler function. This allowed starting with a higher learning rate (0.001), which was then reduced during fine-tuning, resulting in a drastic reduction in the number of epochs needed for model training and, consequently, a lower computational burden, while increasing robustness. The amount of learning rate adjustment was regulated by a factor, γ, that may range from 0 to 1 and was set to 0.6 in this case. Because CNN training is not fully reproducible, due to the random nature of stochastic gradient descent, the performance of models trained on the same data with the same hyperparameters may vary.^41^ To account for this effect, five models were trained consecutively and then tested on an optimization set, consisting of a replicate of the training set image with top polymer height ranging from 0.5 to 9.5 mm, i.e., excluding the pure samples. The model that performed best on the optimization set was selected and used for subsequent prediction steps.

After training, both regression models were used to predict the height of the polymer facing the detector for the internal and external test sets. The predicted heights were then used to generate prediction maps supplemented with the border delineating the actual sample height, as previously described for the CLS approach (Figure 3).

## Results and Discussion

### Spectral Analysis

Initially, the object-based approach was employed to plot and interpret spectral signatures of the two polymers, PETG and PLA, and to identify trends related to polymer height, as illustrated in Figure 4. In particular, the spectra in dark red for PETG-UP and PLA-UP configurations represent the spectrum of the pure polymer (PETG and PLA, respectively), while the other spectra are colored from red to blue according to decreasing height of the upward facing polymer. The analysis of both raw and preprocessed spectra demonstrated consistency between the two instruments, with spectral signatures for HySpex and Specim being almost identical.

**Figure 4:**
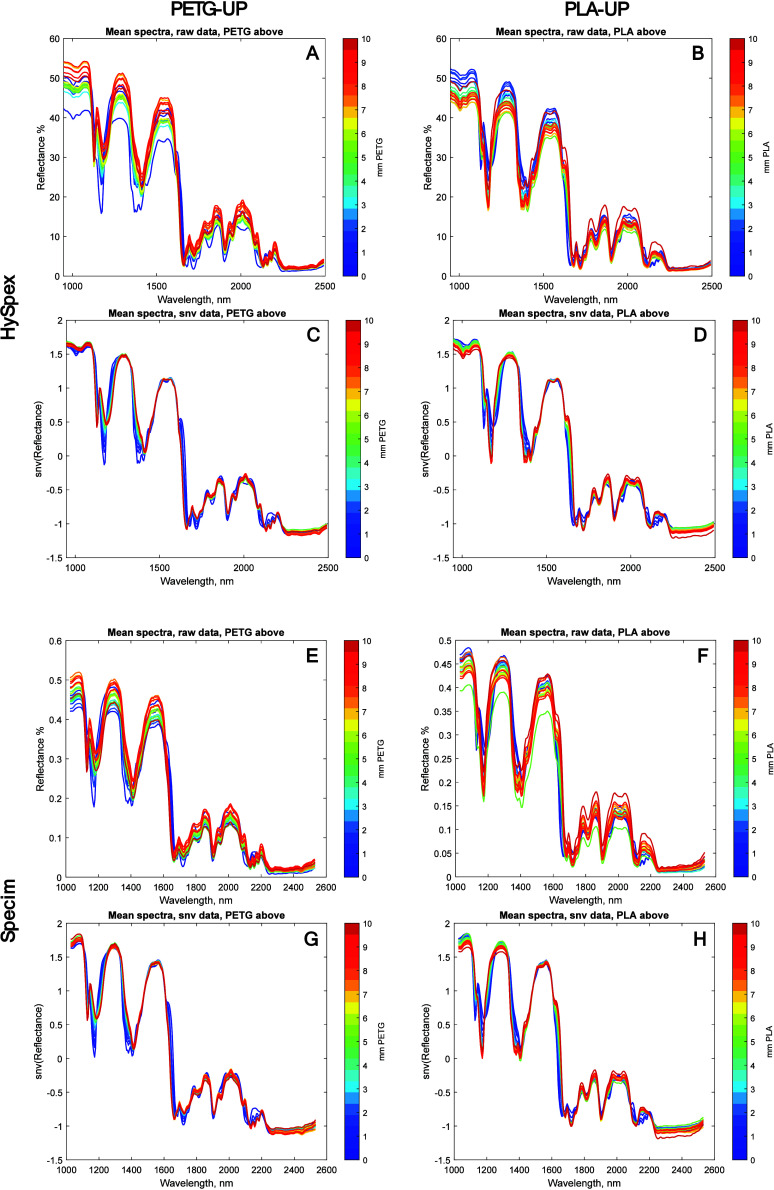
Object-based mean spectra for samples measured with HySpex (from A to D) and Specim (from E to H) systems, colored according to the height of the polymer facing the detector. Spectra appear either in their raw (A-B-E-F) or SNV-transformed (C–D-G-H) form, and the results are reported both for PETG-UP (A-C-E-G) and PLA-UP (B-D–F-H) configurations.

The NIR spectra of the two polymers exhibited pronounced characteristic absorptions within the 1100–1250 nm region. These absorptions were attributed to the second overtone of the carbon–hydrogen (C–H) bond. This region refers to both the antisymmetric methyl and methylene stretching,^44^ which is indicative of the presence of the CH_2_ and CH_3_ groups, and to aromatic C–H stretching. In this region, PLA manifested a single band, likely attributable to the exclusive presence of aliphatic C–H bonds. Conversely, PETG, which presents both aromatic and aliphatic bonds, displayed two bands. A strong band pertaining to the combination of stretching and bending (2ν+δ)^45^ of methyl and methylene groups was discernible at approximately 1400 nm, overlapping with the first overtone of the oxygen–hydrogen bond (O–H) stretching vibration. This band was connected both to the glycol group of PETG and to water in PLA, which is a hygroscopic polymer.^46^ In particular, the humidity contribution for PLA probably outweighed the O–H contribution for PETG, thus rendering PLA absorption more pronounced in this region. Subsequently, from 1600 nm to approximately 2250 nm, the spectra exhibited multiple overlapped absorption bands, whose precise attribution proved complex. This region can be regarded as a fingerprint related to C–H, O–H, C = O and C–O bond vibrations. Conversely, the region of the spectrum after 2250 nm resulted quite flat, exhibiting minimal diagnostic capabilities.

Upon examination of the raw spectra, in Figure 4A,B,E,F, the presence of a total intensity effect emerged. This trend was in part related to the increase in thickness of PLA and to the simultaneous reduction of PETG, with samples with more PLA presenting lower reflectance. This outcome partly confirmed what was evidenced by Pomerantsev et al.,^24,25^ who demonstrated that a reflectance increase can be expected when PE thickness increases. However, in the present case, the relation between increased reflectance and polymer height was not constant nor continuous, therefore this effect was canceled by applying the SNV transform, as illustrated in Figure 4C,D,G,H.

Following application of the SNV transform, a trend related to the height of the polymer facing the detector could be readily discerned. The region between 1100 and 1500 nm showed the greatest variation according to height, with bands indicative of PLA and PETG gradually changing in intensity in accordance with relative amount. A continuous change in these bands was evident for the first half of the samples, where the top polymer gradually increases from 0 to approximately 5 mm (from blue to aquamarine in the figure). In the second half, going from 5 to 10 mm (from aquamarine to red in the figure), the spectra of subsequent ROIs overlapped significantly. The region between 2100 and 2250 nm was of particular interest, as it allowed distinguishing which of the two polymers was facing the detector. Indeed, in the PETG-UP configuration, this region maintained a more defined shape, characterized by three narrow, consecutive bands. On the contrary, in the PLA-UP configuration the region kept a broader and rounder shape, with the distinct bands being discernible only at very low heights of PLA. This finding indicated that different radiation-interaction behaviors might depend on the orientation of the polymer. It is also noteworthy that, after application of the SNV transform, the area around 2200–2400 becomes more related to polymer height, showing some correlation also for samples in the 5–10 mm range. This may be a consequence of the preprocessing applied, since SNV is known for pushing variability out to the edges of spectral data.^47,48^

### PCA

Subsequently, exploratory analysis by means of PCA was carried out, and the results are illustrated in Figure 5. The application of Savitzky-Golay first derivative followed by SNV transform resulted to be the best choice to ensure that the highest source of variability, connected to the information of interest, i.e., height proportion, could be condensed in PC1, having minimized the extent of unwanted systematic variations, ascribable to physical properties of the specimens. This preprocessing was then applied to all subsequent processing steps, with the exception of CNN.

**Figure 5:**
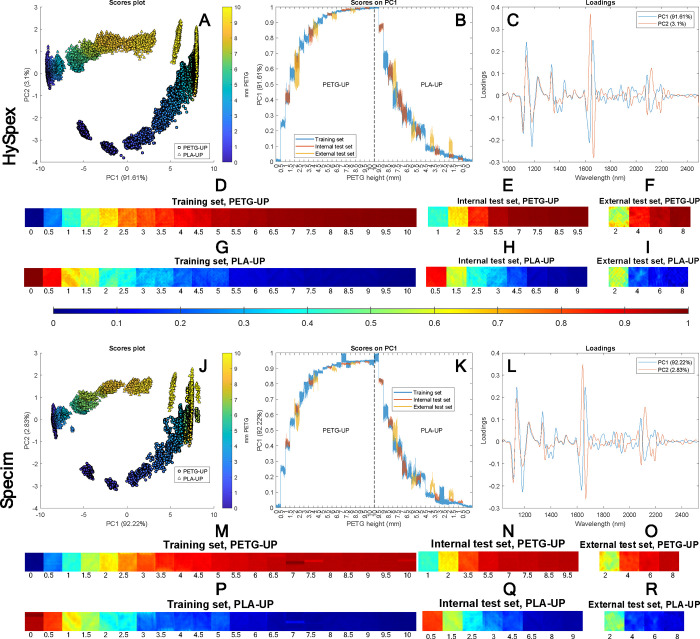
PCA results for HySpex (from A to I) and Specim (from J to R) data. In particular, score plots colored by PETG height (A-J), score value for PC1 against height of PETG (B–K), loading plots (C-L), score maps of the training set PETG-UP (D-M), internal test set PETG-UP (E-N), external test set PETG-UP (F–O), training set PLA-UP (G-P), internal test set PLA-UP (H-Q) and external test set PLA-UP (I-R) are reported. Numbers under the score maps represent the height in mm of the polymer-up associated with the corresponding ROI.

When considering the score plots colored according to the height in mm of PETG, displayed in Figure 5A,J, it was indeed clear that PC1, which explained more than 90% of the total variance, summarized the information regarding polymer height, as clearly underlined by the change of color from blue to yellow moving from low to high scores values on PC1. Additionally, the score plot also showed that PC2 explained the spectral differences related to the specific polymer facing the detector. In particular, samples in the PLA-UP configuration, represented by triangles in the plot, were generally located at positive scores on PC2, while samples in the PETG-UP configuration, represented by circles, could be found at negative values of this component. Afterward, the analysis focused on PC1 score values, which were plotted in relation to the height of the polymer facing the detector in Figure 5B,K, evidencing a clear nonlinear behavior. For PETG-UP, a pronounced change was evident at the beginning of the trend, with substantial variations when the height of the polymer facing the detector changed from 0 to approximately 3 mm, diminishing variations in the range of 3 to 6.5 mm, and a stabilization of score values subsequently. Similarly, PLA-UP scores demonstrated significant alterations from 0 to approximately 6 mm, with a rapid stabilization of score values thereafter. This trend was visually confirmed by the score maps of the training set for both PETG-UP (Figure 5D,M) and PLA-UP (Figure 5G,P): while the first seven ROIs rapidly changed color from blue to red or vice versa, the remaining 14 samples slowly became darker, with the last ROIs being almost indiscernible. This behavior mirrors what was already noticed in the analysis of raw and preprocessed spectra.

The chemical nature of the information retained in PC1 was confirmed by the loading plots (Figure 5B,G,L,Q), which presented high values in the 1100–1250 nm region, corresponding to the second overtone of C–H vibration, as previously identified. The areas of greatest importance in the loadings corresponded to the region exhibiting the most significant variations in the spectra, lending further support to the hypothesis that variations in polymer height were indeed the variation captured by PC1. In addition, the spectral region between 1600 and 1700 nm showed high values in the loading plots. Though it is imperative to exercise caution when evaluating band attribution in preprocessed data,^48^ a re-examination of the raw data profiles (Figure 4A,B,E,F) revealed that the main difference between the two polymers in this region could be ascribed to a shoulder feature characteristic of PLA. This result is likely attributable to the C–H first-overtone (2ν)^45^ contribution from the stretching of the methyl group. Indeed, PLA structure presents a pendant −CH_3_ group absent in PETG, and the presence of a chiral backbone in PLA may therefore engender a distinct C–H environment compared to that observed in PETG’s glycol/ethylene units, likely resulting in the formation of this highly diagnostic extra shoulder.

It was noteworthy that, despite retaining both orientations in the model calibration set, the main source of variability was the height-proportion between the two polymers. This finding emphasized the efficacy of PCA in discerning chemical information from the samples without being confounded by their orientation. To further substantiate this result, the PCA modeling was repeated also making two separate models for the two configurations. The results of this additional analysis are displayed in Figure S1 of the Supporting Information, where both the PC1 vs top/polymer height and the loading plots demonstrated a high degree of similarity across the various models, both when compared with Figure 5 and to each other. In particular, all loading plots were almost identical, demonstrating a strong robustness of the approach.

Finally, the pixel spectra from the internal and external test sets were projected into the PC space of the training set, and the resulting scores were analyzed.

The internal test set results exhibited substantial correspondence with the training set, as can be observed in Figure 5B,K, where the scores of the internal test set (in red) completely overlap with the training set values (in blue). The noise levels of the two sets were comparable as well. This was also confirmed by the uniform aspect of the maps (Figure 5E,H,N,Q), which did not display any systematic variation, and showed consistency in the color associated with each height when compared with the training set results. Additionally, a flattening at increasing top-polymer height was evident.

However, successive analysis of the external test set scores revealed discrepancies when compared to the training set. In Figure 5B,K, external test set scores (in yellow) presented increased noise and were not perfectly overlapped with the training set. The source of the increased noise could be detected through observation of the score maps (Figure 5F,I,O,R) as attributable to the use of an alternative infill pattern during specimen production, which could be readily distinguished by visual inspection of the score maps. In particular, when observing the HySpex results for the samples using the Hilbert Curve infill pattern (Figure 5E,J, first and last ROI of the series), the pattern could clearly be identified. Indeed, the printing pattern used for the external test set was different from the training set, therefore this source of variability reappeared in the scores. Furthermore, the external test set score maps revealed color differences when moving from 6 to 8 mm in the PETG-UP configuration, which were indiscernible in the training set, suggesting higher radiation penetration in the external test set samples. This could also be observed as a shift toward lower PC1 score values in Figure 5B,K. This result confirmed that the infill pattern, modifying the internal structure of the sample, influenced the penetration depth of the NIR radiation, indicating that the process may be strongly influenced by the physical properties of the sample.

When the results from HySpex (Figure 5A–I) and Specim (Figure 5J–R) were compared, they were once again found to be highly similar, with almost identical score maps and comparable amounts of explained variance in the first component. However, in the Specim scores, the printing pattern in the external test set was less evident, probably due to the instrumental setup, which resulted in lower spatial resolution and consequently a higher pixel size.

In summary, PCA was valuable in confirming, to some extent, the penetration of NIR-SI into matter, showing a relationship between the PC1 score values and the top polymer height proportion. However, the identification of a penetration limit proved to be quite challenging. The nonlinear behavior observed in the scores may indeed be attributable to a reduced penetration in the deeper layers, but it is also possible that increased dominance of scattering phenomena, due to the radiation having to cross an increasing number of printing layers, may be responsible for the observed effect. The mentioned phenomena may confound the chemical information, thereby reducing the macroscopic differences between successive samples, even if the penetration remains effective. Consequently, further chemometric analysis was required to explore this topic more thoroughly. In particular, while PCA provided an overview of the main sources of spectral variance and suggested a potential relationship with height proportion, a quantitative approach was required to decompose the spectra into contributions from individual polymer components. To this end, CLS was applied to directly assess the relative contributions of the top and bottom layers.

### CLS

CLS was therefore employed as a signal deconvolution technique to derive pixel-based predicted heights of the components using the spectral signatures of the pure components. The results of the deconvolution process are presented in Figure 6. As demonstrated in the predicted versus measured plot (Figure 6A,E,I,M), where the predicted value for each ROI is displayed as the average of its pixel predictions, all the sets exhibited a nonlinear trend, in accordance with the observations made from PC1 scores.

**Figure 6:**
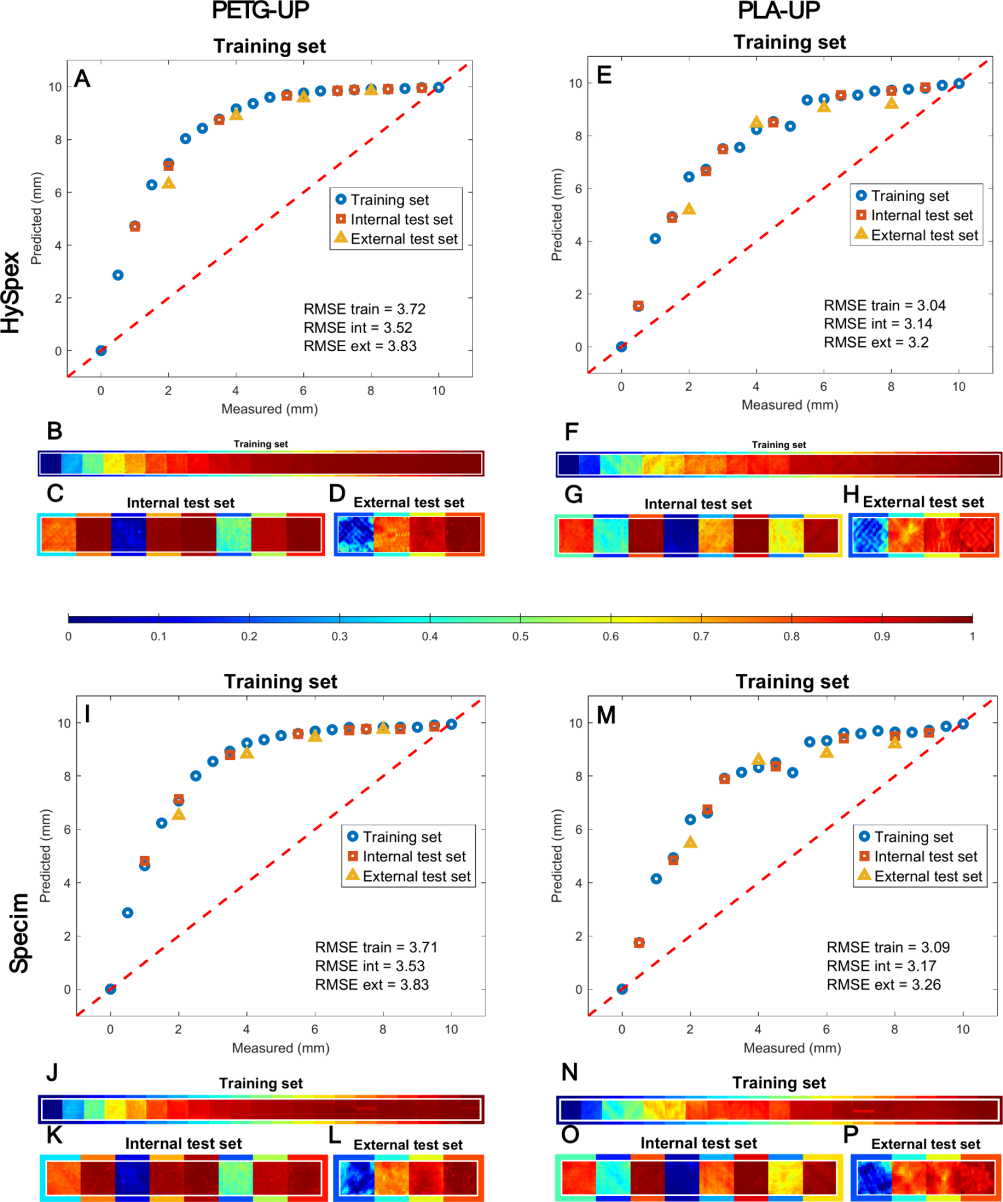
CLS results for HySpex (from A to H) and Specim (from I to P) data for PETG-UP (A–B-C-G–H-I) or PLA-UP (D-E-F-J-K-L) configuration. In particular, predicted versus measured plots (A, E, I, M), prediction maps for the training set (B–F-J-N), for the internal test set (C-G–K-O), and for the external test set (D-H-L-P) are displayed. Each prediction map is reported with a border which represents the color the corresponding ROI should have if its prediction error was null.

However, some discrepancies were observed between the two materials. In particular, the RMSE of the PLA-UP configuration, though still unacceptable for modeling purposes, was reduced by 0.6 mm on average when compared to the PETG-UP configuration. This may indicate that the penetration of NIR radiation in PLA is greater than in PETG. Indeed, in the PLA-UP configuration samples in the 6–10 mm height range still presented a slight increasing trend despite the flattening, as evident in Figure 6E,M. This could not be observed in the PETG-UP configuration. This finding aligns with the established literature on the subject, which posits a correlation between penetration and chemical properties.^4^

Afterward, CLS was employed to generate prediction maps of the top-polymer height for all sets. A comparison of the color of each ROI with its border, representing the actual sample height – i.e., the color that the ROI should have if the prediction had no error – indicated that the height of the polymer facing the detector was overestimated across the entire range. Furthermore, an increased level of noise was evident in the external test set samples, attributable to the different printing pattern. This observation confirmed the findings reported in PCA. Moreover, the results obtained with the two instruments were highly similar, thereby confirming the robustness and reproducibility of the approach.

To reduce the nonlinear shape in the plots and evidence changes even at higher polymer height, both PCA scores and CLS estimated values were plotted against the logarithm of the top-polymer height in Figure S2 of the Supporting Information. This representation successfully reduced the nonlinearity of the plot for both polymers; however, it did not solve it completely. This transformation was instrumental in proving a pseudologarithmic behavior for the radiation penetration.

The use of CLS for deconvolution substantiated the notion that NIR radiation is indeed capable of penetrating the matter, as already underlined by PCA. It confirmed that the penetration of NIR-SI was characterized by nonlinear behavior with respect to component height, which, even after a logarithmic transformation of sample height, could not be accurately accounted for by unsupervised chemometric strategies, that do not take this additional information into account during model calibration. The nonlinear behavior observed for CLS estimated values, as well as for PCA scores, might be seen, at first glance, as analogous to an approximate Beer–Lambert-type trend. However, since the present measurements were performed in the reflection mode on a solid system, and the optical path length through the analyte layer was itself variable, deviations from an ideal linear behavior, as observed here, are expected. Additionally, to confirm that the nonlinearity observed was indeed intrinsic to the studied phenomenon, the CLS calculations were repeated converting the spectral data to the pseudoabsorbance by calculating -Log­(R). The results, reported in Figure S3 of the Supporting Information, displayed the same behavior observed in Figure 6, therefore confirming a nonlinearity in the penetration process, likely connected to the large path length.

### Regression

Regression was employed to determine whether the flattening tendency observed in PCA and PLS could indeed be attributed to a lack of penetration after approximately 6 mm, or rather if, directing the model toward the desired information by means of a response variable, scattering and other confounding effects could be successfully neglected, enhancing the chemical information derived from the deepest layers. It is important to note that regression techniques were not utilized to obtain a robust prediction model to be applied in real-world applications. Instead, these techniques were exploited to ascertain whether precise and accurate predictions could be made, with the assumption that an ability to achieve both precise and accurate predictions must be the consequence of NIR radiation penetration to the considered depth.

#### PLS

First, a PLS regression model was calibrated on the training set. The number of selected latent variables for each model is reported in Table 1, while the predicted versus measured plot for the training set and its prediction image are reported in Figure S4 of the Supporting Information. Model training produced slightly better results for the PLA-UP configuration than the PETG-UP one, with lower RMSECV, consistent with the CLS results above. This finding corroborated the earlier hypothesis of a deeper penetration in PLA compared to PETG, thereby sustaining that NIR radiation penetration is material dependent. Furthermore, while the PLA-UP model exhibited a linear trend along the entire calibration range (Figure S4C,G), PETG-UP presented a flattening when the polymer height exceeded 7 mm (Figure S4A,E). This trend, though less pronounced, confirms the findings of PCA and CLS, suggesting that penetration in PETG may indeed be limited at approximately 7 mm. In contrast, PLS regression could extract information from the deepest layers of samples in the PLA-UP configuration. The reduced or absent flattening identified during PLS regression provided support for the hypothesis that at least some of the nonlinearity observed with the unsupervised methods resulted from scattering phenomena that could be resolved by the PLS regression approach.

**Table 1:** Summary of the Statistics Calculated for the PLA and CNN Models for Training Set, Cross-Validation, Internal Test Set and External Test Set^a^

			**PETG-up**				**PLA-up**			
			*training set*	*cross-validation*	*internal test set*	*external test set*	*training set*	*cross-validation*	*internal test set*	*external test set*
**HySpex**	**PLS**	*RMSE*	0.64	0.77	0.79	0.56	0.52	0.68	0.49	0.56
		*bias*	0	0.02	0.31	–0.05	0	0.004	0.10	–0.16
		*LVs*	6	-	-	-	5	-	-	-
	**CNN**	*RMSE*	0.18	-	0.24	0.72	0.14	-	0.16	0.70
		*bias*	0.002	-	0.01	–0.58	–0.01	-	0.01	–0.52
**Specim**	**PLS**	*RMSE*	0.62	1.08	0.94	0.84	0.53	0.75	0.55	0.76
		*bias*	0	–0.10	–0.16	–0.30	0	0.02	0.07	–0.26
		*LVs*	10	-	-	-	7	-	-	-
	**CNN**	*RMSE*	0.16	-	0.73	0.50	0.12	-	0.60	1.04
		*bias*	0.007	-	–0.56	–0.20	0	-	–0.35	–0.78

a The models for either PETG-UP or PLA-UP are reported. RMSE and bias values are reported in mm.

When examining the VIP scores derived from the PLS regression, reported in Figure 7A,C,E,G, it was possible to identify some differences between the descriptors involved in the model for PETG-UP (Figure 7A,E) when compared to the ones for PLA-UP (Figure 7C and **G**). This finding was in disagreement with the PCA loadings, which exhibited nearly identical shapes for the two polymers. In particular, a region around 1350 nm showed higher VIP scores for describing penetration through PETG, while the 1600–1700 nm range had slightly higher weight in the PLA-UP model. Additionally, a contribution from the 2050–2150 nm range, of slightly greater significance for PETG-UP, could now be identified. These observations were strongly consistent between instruments, with indiscernible VIP profiles for the HySpex and Specim apparatuses, supporting the robustness of the conclusions.

**Figure 7:**
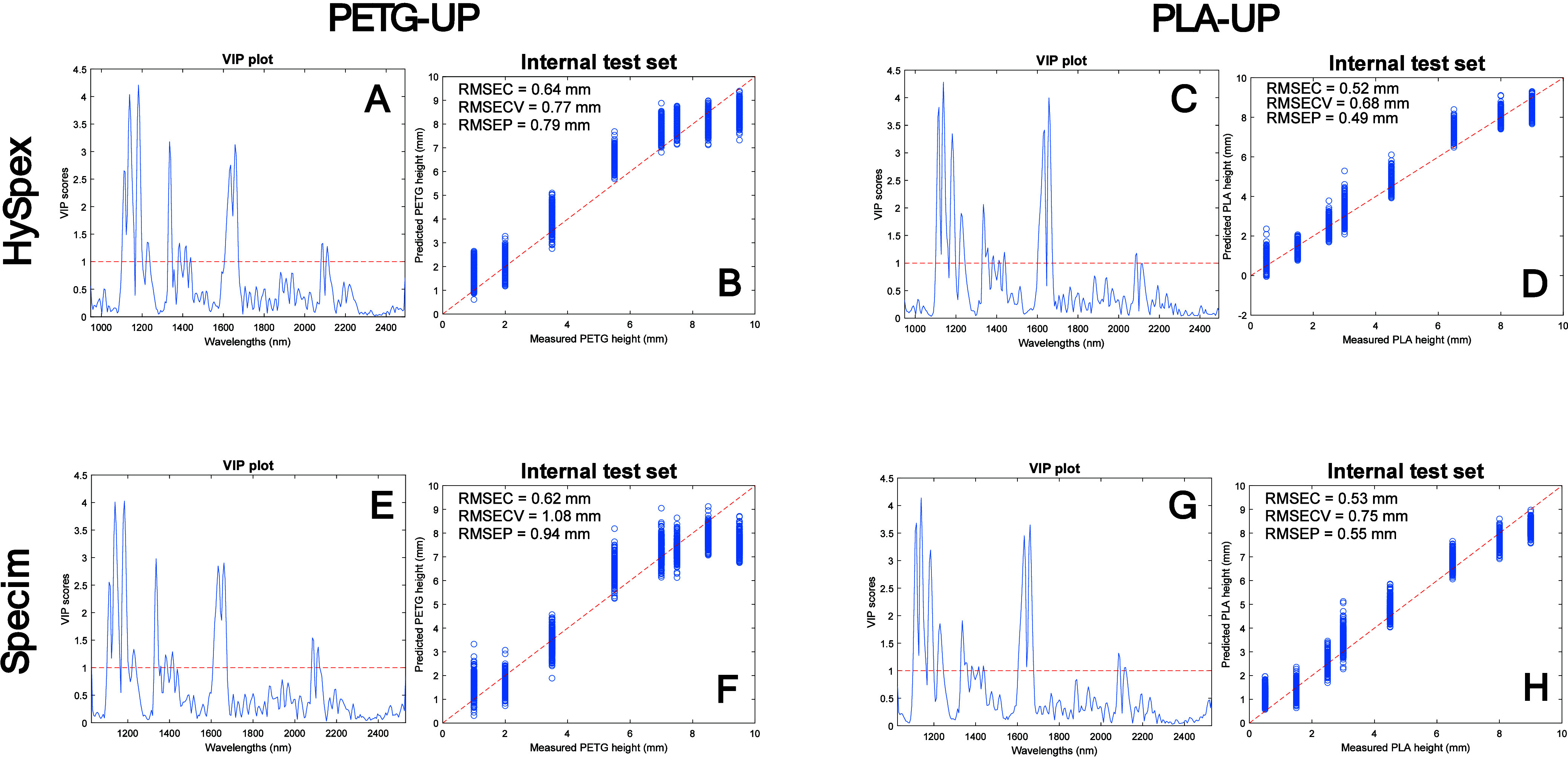
PLS regression results for HySpex (A-B-C-D) and Specim (E-F-G-H) data for PETG-UP (A-B-E-F) or PLA-UP (C-D-G-H). In particular, the VIP plots (A-C-E-G) and the predicted versus measured plots for the internal test set (B-D-F-H) are displayed.

The findings from the training set were subsequently corroborated when the internal and external test sets were projected into the calibration space. In particular, when the predicted versus measured plot for the internal test set was considered (Figure 7B,D,F,H), the expected flattening could be observed for PETG-UP, while this was not evident for PLA-UP. In general, predictions for both the test sets could be regarded as satisfactory, as sustained by the RMSEPs reported in Table 1. This was also supported by the prediction maps in Figure 8, which showed a good match between the border color and the ROI color. Moreover, the internal test set presented lower noise than the external test set, as evidenced by the grainier appearance of the latter.

**Figure 8:**
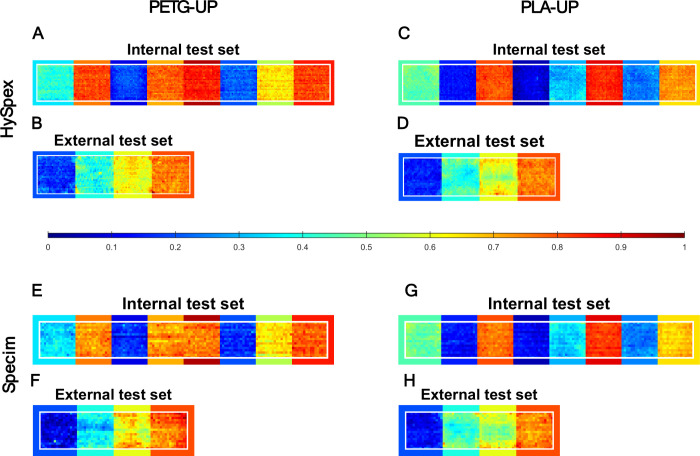
PLS prediction results for HySpex (A–B-C–D) and Specim (E-F-G-H) data for PETG-UP (A-B-E-F) or PLA-UP (C–D-G-H). In particular, the prediction maps for the internal test set (A-C-E-G) and for the external test set (B-D–F-H) are displayed. Each prediction map is reported with a border which represents the color the corresponding ROI should have if its prediction error was null.

However, the RMSEP behavior exhibited differences between PETG-UP and PLA-UP. In PETG-UP, RMSEP was higher for the internal test set compared to the external test set, while this behavior was reversed for PLA. This finding may be connected to a higher penetration through the external test set samples due to printing pattern, as previously hypothesized from CLS results. As a consequence, for PETG-UP the higher penetration of radiation through the external test set samples may have corrected for the flattening observed in the training and internal sets, thus improving the prediction. Conversely, for PLA-UP the heightened penetration of NIR radiation through the external test set samples became inconsequential, as this polymer presented good penetration capabilities, i.e., linearity, along the whole range, as previously highlighted. In this particular instance, the different printing pattern of the external test set may have been responsible for the increased noise, without gaining much in terms of penetration and linear fitting, thus increasing the prediction error. These observations provided further evidence for the hypothesis that NIR radiation penetration is dependent on complex physical phenomena in addition to chemical information.

Additionally, some discrepancies between the results obtained from Specim and HySpex were revealed in PLS. In particular, while the aforementioned similarities and differences remain constant, the values of RMSE proved to be lower for the HySpex-based models compared to the Specim ones, as evidenced in Table 1. Furthermore, Specim-based models were characterized by increased levels of complexity (6 and 5 LVs versus 10 and 7). However, these different performances were likely not attributable to an instrumental disparity, but rather to the different lamp intensity levels employed in the two instrumental setups. Indeed, the lamps coupled to the HySpex apparatus presented higher power compared to the ones coupled to the Specim one, thus suggesting that the stronger illumination may cause increased penetration, as indicated by the improved performances of the models. This comparison allows highlighting that the illumination setup can play a crucial role in optimizing the penetration of the radiation, also depending on the specific application.

However, PLS regression was deemed insufficient to completely rule out the possibility that NIR radiation was penetrating PETG across the entire range. Indeed, since PLS regression is a linear method, it cannot fully account for nonlinear behavior in the data. The application of a nonlinear regression technique may therefore be indicated to determine whether penetration through PETG also occurs. Consequently, CNN were implemented to capture complex spectral-structural interactions and to improve predictive accuracy further.

#### CNN

Finally, the data were preprocessed by means of SNV transform and a CNN model was calibrated on the training set pixel spectra. The training results are reported in Figure S5 of the Supporting Information and in Table 1. When the predicted versus measured plots of the models referred to PETG-UP (Figure S5A,E) were observed, the flattening trend was no longer evident. Training performances for the two polymers were now comparable, thus suggesting that CNN, given its capability to deal with nonlinearity, was able to adequately describe penetration in both materials. The goodness of the training process can also be substantiated by the prediction maps (Figure S5B,D,F,H), which demonstrate an almost perfect congruence between the ROI predicted color and the expected value, as represented by the border.

Also in this case, the outcomes derived from the projection of the internal and external test sets onto the calibration space corroborated the observations made on the training set, as illustrated in Figure 9. Indeed, both the internal and external test sets showed a satisfactory alignment with the regression line, without discernible flattening. Interestingly, however, some heteroskedasticity could be observed, with error increasing with polymer height, suggesting a progressive deterioration of model performances with increasing penetration depths. Moreover, the internal test set consistently exhibited superior performances in comparison to the external test set. This outcome suggested that the differences in printing patterns played a substantial role in the prediction process. Furthermore, these models also demonstrated a moderate degree of overfitting, with the model exhibiting an RMSEC that was almost four times lower than the RMSEP_EXT_. This outcome may be at least in part connected to the use of pixels as individual samples for model training. Indeed, while this approach is mandatory for CNN training, which requires a high amount of data to be robustly calibrated, it may introduce a certain degree of overfitting in the resulting models due to the high amount of duplicated samples. As a result, the model was highly accurate and precise in describing samples similar to those in the training set, including those from the internal test set. However, its limitations became evident when describing samples with distinct physical characteristics, such as those forming the external test set, which generally presented increased RMSEP and bias when compared to the PLS model. Additionally, an analysis of the predicted versus measured plots of the external test set (Figure 9B,D,F,H) revealed a systematic underestimation of top-polymer height by the model.

**Figure 9:**
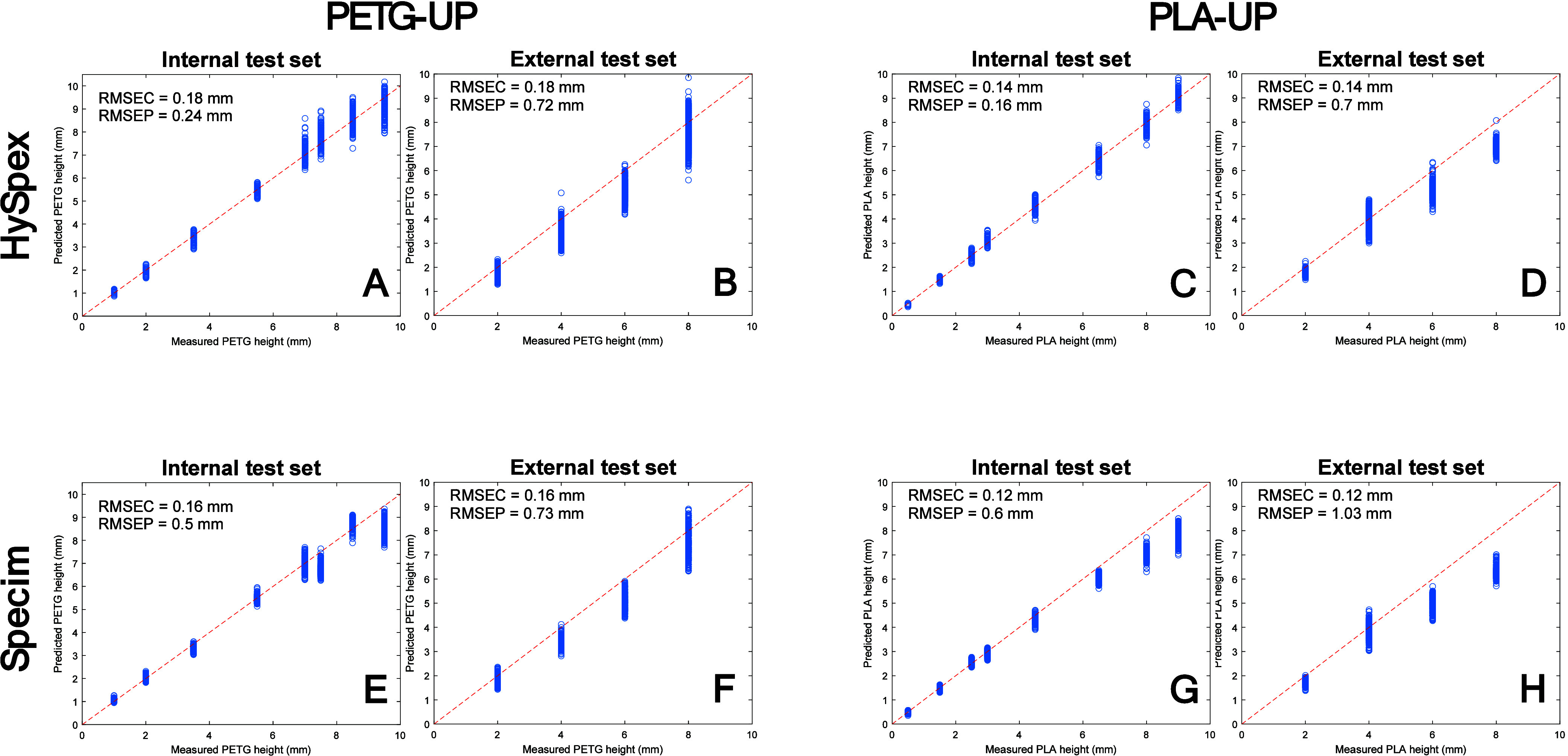
CNN regression results for HySpex (A–B-C–D) and Specim (E-F-G-H) data for PETG-UP (A-B-E-F) or PLA-UP (C–D-G-H). In particular, the predicted versus measured plots for the internal test set (A-C-E-G) and the external test set (B-D–F-H) are displayed.

An observation of the prediction maps, displayed in Figure 10, confirmed the differences between internal and external test sets already noted through observation of Figure 9. In particular, while an almost perfect match between the border and the ROI colors was observed in the internal test set maps, a systematic difference of the ROI color could be observed for the last two samples of the external test set, in particular for the PLA-UP configuration. Compared to the prediction maps obtained from the PLS regression (Figure 8), those obtained from CNN were characterized by a reduction of random noise, as demonstrated by the lower grainy aspect of the ROIs. On the other hand, the CNN prediction maps obtained for the external test set were characterized by an evident contribution of the printing pattern that could be visualized in the prediction images, demonstrating a higher influence of physical features on the models.

**Figure 10:**
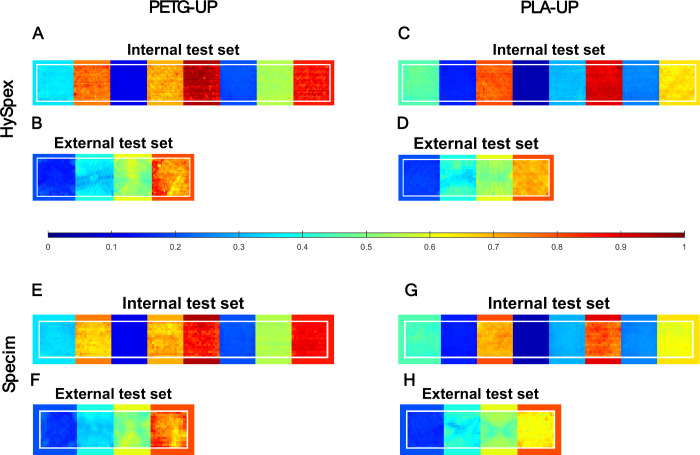
CNN prediction results for HySpex (A–B-C–D) and Specim (E-F-G-H) data for PETG-UP (A-B-E-F) or PLA-UP (C–D-G-H). In particular, the prediction maps for the internal test set (A-C-E-G) and for the external test set (B-D–F-H) are displayed. Each prediction map is reported with a border which represents the color the corresponding ROI should have if its prediction error was null.

A comparison between the polymers was challenging, since some discrepancies could be observed between HySpex and Specim results. While the two instruments demonstrated coherent performances for the PETG-UP configuration, the performances for PLA-UP were significantly worse when assessed by means of the Specim apparatus. This outcome might again be ascribable to the different lamp intensities of the two settings.

In summary, the use of CNN was instrumental for demonstrating the capability of NIR radiation to penetrate through at least 1 cm in both the studied polymers, confirming that the phenomenon was characterized by a complex behavior entailing a nonlinear trend in the deepest layers. Indeed, the models, though slightly overfitted and biased on the external test set, enabled the resolution of the flattening observed in all the chemometric approaches previously applied, suggesting that, with the help of appropriate nonlinear methods, penetration may be described even in deeper layers.

## Conclusions

The present study establishes a comprehensive framework for the quantitative assessment of NIR radiation penetration in SI by implementing appropriate chemometric approaches to disentangle and model subsurface information. By integrating PCA, CLS, and PLS regression with CNN, the research demonstrates that spectral signals can be detected from layers located up to approximately 1 cm beneath the surface in both PETG and PLA. Importantly, penetration depth was confirmed to depend strongly on the interplay between material-specific optical properties (chemical composition, morphology, printing pattern) and instrumental parameters (illumination intensity), rather than being a fixed characteristic. Moreover, CNN-based models, in particular, revealed a nonlinear penetration behavior, confirming the complexity of the scenario and extending the information derived from linear methods.

Rather than defining a fixed penetration limit, the present framework provides a quantitative methodology for assessing how NIR radiation interacts with layered media. The results highlight that NIR-SI spectra may include contributions from layers significantly deeper than usually assumed, sometimes exceeding 1 cm, emphasizing the need for careful data interpretation and spectral unmixing in real-world applications.

Looking ahead, applying this framework to thicker and more complex multicomponent samples will be essential to delineate the ultimate boundaries of NIR-SI penetration and to further explore the nonlinear interplay between optical, structural, and instrumental variables. Additionally, systematic evaluation of wavelength-dependent effects will shed light on how different energy regions influence penetration and information retrieval.

Taken together, the present study redefines the conventional understanding of NIR-SI as a surface analysis method, demonstrating its potential to evolve into a depth-resolved analytical and tomographic tool. The proposed framework for nondestructive, material-specific analysis of penetration for complex samples paves the way for novel applications of NIR-SI in quality control, materials design, and nondestructive testing of complex multilayer systems, thereby setting the stage for the next generation of spectral imaging methodologies.

## Supplementary Material



## Notes

Table S1, detailing the characteristics of samples present in training, internal test and external test sets; Figure S1 displaying the predicted versus measured plots for the CLS models; Figure S2 displaying the PLS regression predicted versus measured plots and the prediction maps for the training set; Figure S3 displaying the CNN regression predicted versus measured plots and the prediction maps for the training set (PDF)
