# Supplementary material for: What Lies INSIDE: Chemometric Insights on the Penetration Depth of Near-Infrared Radiation in Spectral Imaging Configurations
Source: Anal Chem. 2026 Jun 25;98(26):19567–81. doi: 10.1021/acs.analchem.6c00848 (PMC13347703; doi:10.1021/acs.analchem.6c00848)
Supplement: Supplementary file 1 [file ac6c00848_si_001.pdf]

What lies INSIDE: chemometric insights on the penetration depth of near infrared radiation in spectral imaging configurations

Sara Gariglio[a,b], Cristina Malegori[a], Paolo Oliveri[a,*], Monica Casale[a], Carolina Scagliarini[c], Alberto Mazzoleni[c], Eugenio Alladio[c], Zelan Li[d], Emilio Catelli[d], Giorgia Sciutto[d], Aoife Gowen[e], Sergey Kucheryavskiy[f]

[a] *Department of Pharmacy (DIFAR), University of Genova, Viale Cembrano 4, Genova, 16148, Italy*
[b] *Department of Chemistry and Industrial Chemistry (DCCI), University of Genova, Via Dodecaneso 31, Genova, 16146, Italy*
[c] *Department of Chemistry, University of Torino, Via Pietro Giuria 7, Torino, 10125, Italy*
[d] *Department of Chemistry "Giacomo Ciamician", University of Bologna, Via Guaccimanni 42, Ravenna, 48121, Italy*
[e] *School of Biosystems and Engineering, University College Dublin, Belfield, Dublin 4, D04 V1W8, Ireland*
[f] *Department of Chemistry and Bioscience, Aalborg University, Niels Bohrs Vej 7, Esbjerg, 6700, Denmark*
**Corresponding Author:* paolo.oliveri@unige.it

TABLE OF CONTENTS

Table S1 .... page S2
Figure S1 .... page S3
Figure S2 .... page S3
Figure S3 .... page S4
Figure S4 .... page S5
Figure S5 .... page S6

	Sample type	Number of samples	Sample height (PLA-PETG mm)	ROI size		Total number of pixels	
				Specim	***HySpex***	***Specim***	***HySpex***
Training set	Cubes	21	0-10, 0.5-9.5, 1-9, 1.5-8.5, 2-8, 2.5-7.5, 3-7, 3.5-6.5, 4-6, 4.5-5.5, 5-5, 5.5-4.5, 6-4, 6.5-3.5, 7-3, 7.5-2.5, 8-2, 8.5-1.5, 9-1, 9.5-0.5, 10-0	16×16	26×26	5376	14196
Internal test set	Cubes	8	4.5-5.5, 1.5-8.5, 8-2, 0.5-9.5, 3-7, 9-1, 2.5-7.5, 6.5-3.5	16×16	26×26	2048	5408
External test set	Cylinders	4	2-8, 4-6, 6-4, 8-2	16×16	22×22	1024	1936

***Table S1**: Details of sample shapes and heights, sample numbers, polymer heights, ROI shape and matrix dimension for training set, internal test set and external test set.*

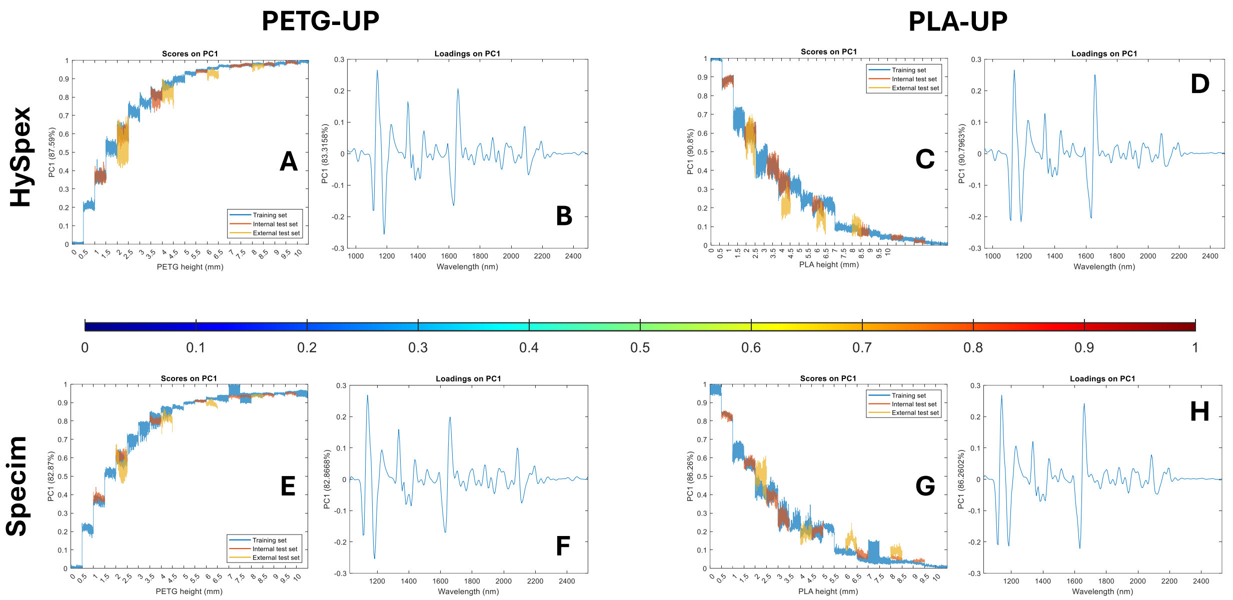

***Figure S1**: PCA results for HySpex (from A to D) and Specim (from E to H) data for PETG-UP (A-B-E-F) and PLA-UP (C-D-G-H) configurations. In particular, score value for PC1 against height of the polymer facing the detector (A-C-E-G) and loading plots (B-D-F-H), are reported.*

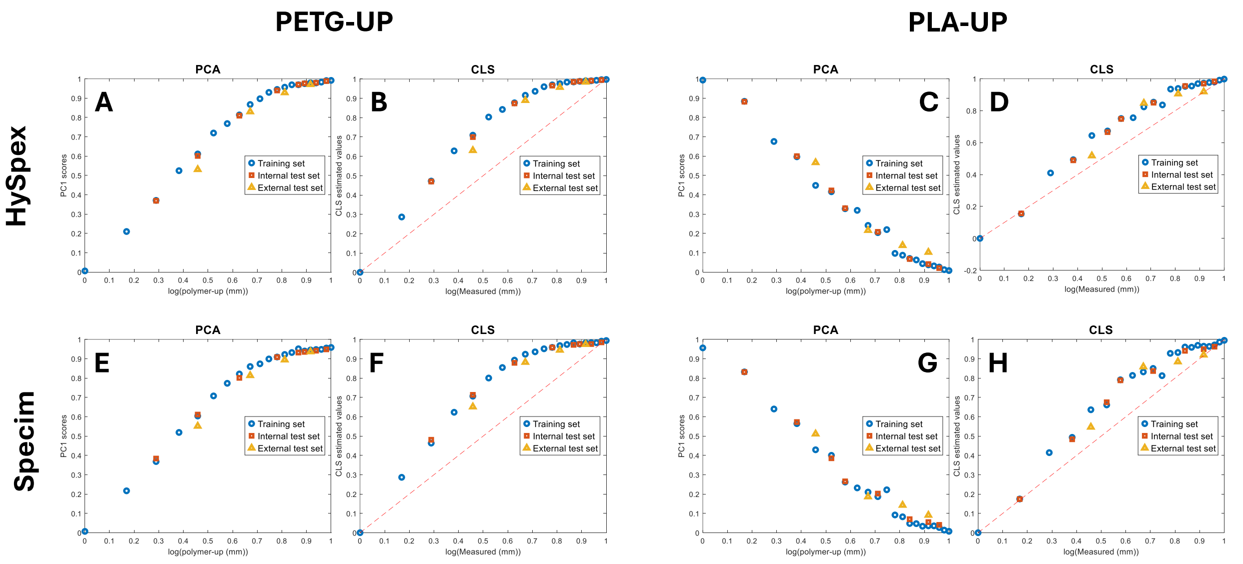

***Figure S2**: PCA scores and CLS predicted values plotted against the logarithm of polymer-up height in mm for HySpex (A-B-C-D) and Specim (E-F-G-H) data for PETG-UP (A-B-E-F) or PLA-UP (C-D-G-H) configuration. In particular, PCA scores (A, C, E, G) and CLS predicted values (B, D, F, H) versus the logarithm of measured polymer height are displayed.*

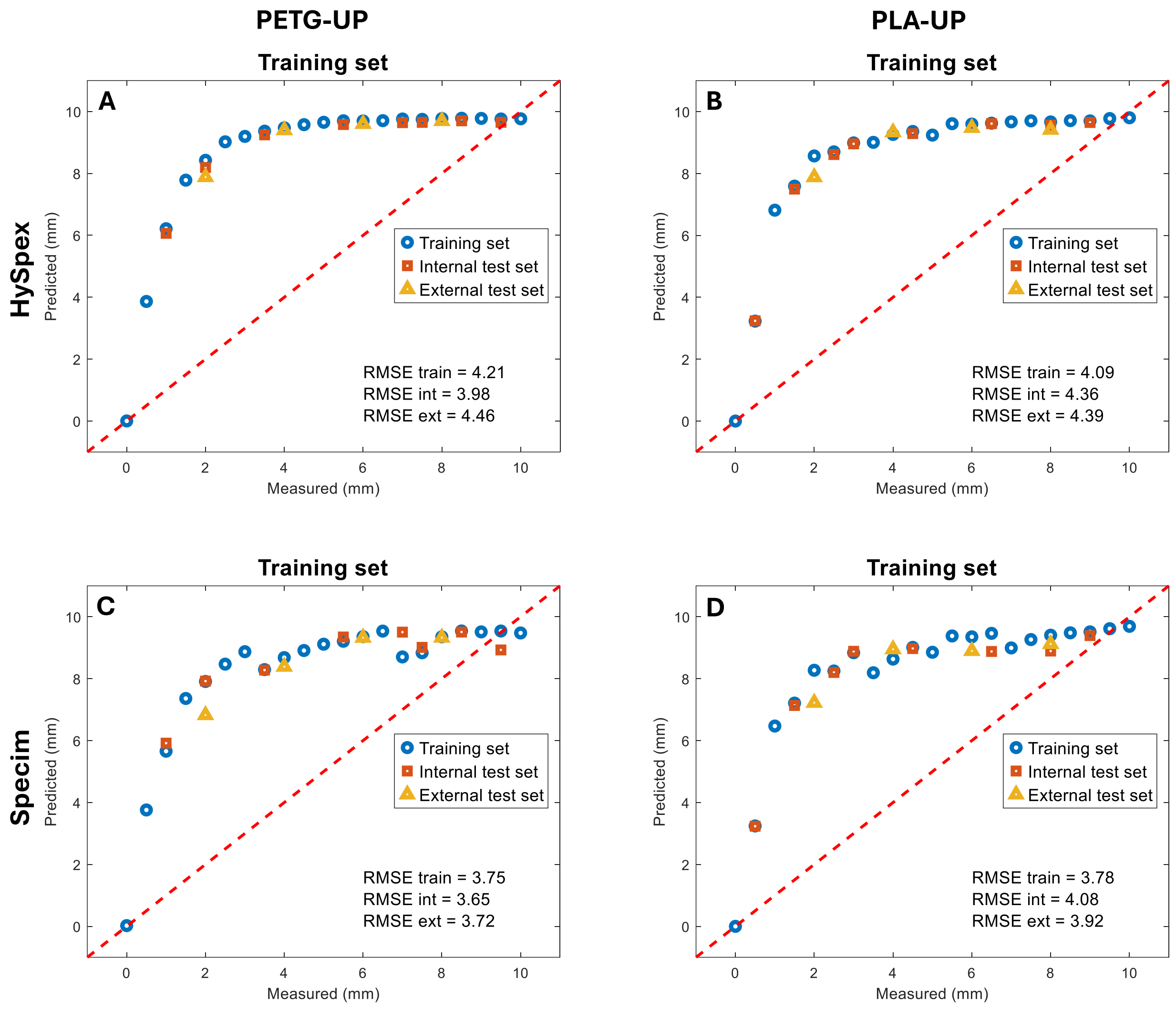

Figure S3*: PCA and CLS scores plotted against the logarithm of polymer-up height in mm for HySpex (A-B) and Specim (C-D) data for PETG-UP (A-C) or PLA-UP (B-D) configuration.*

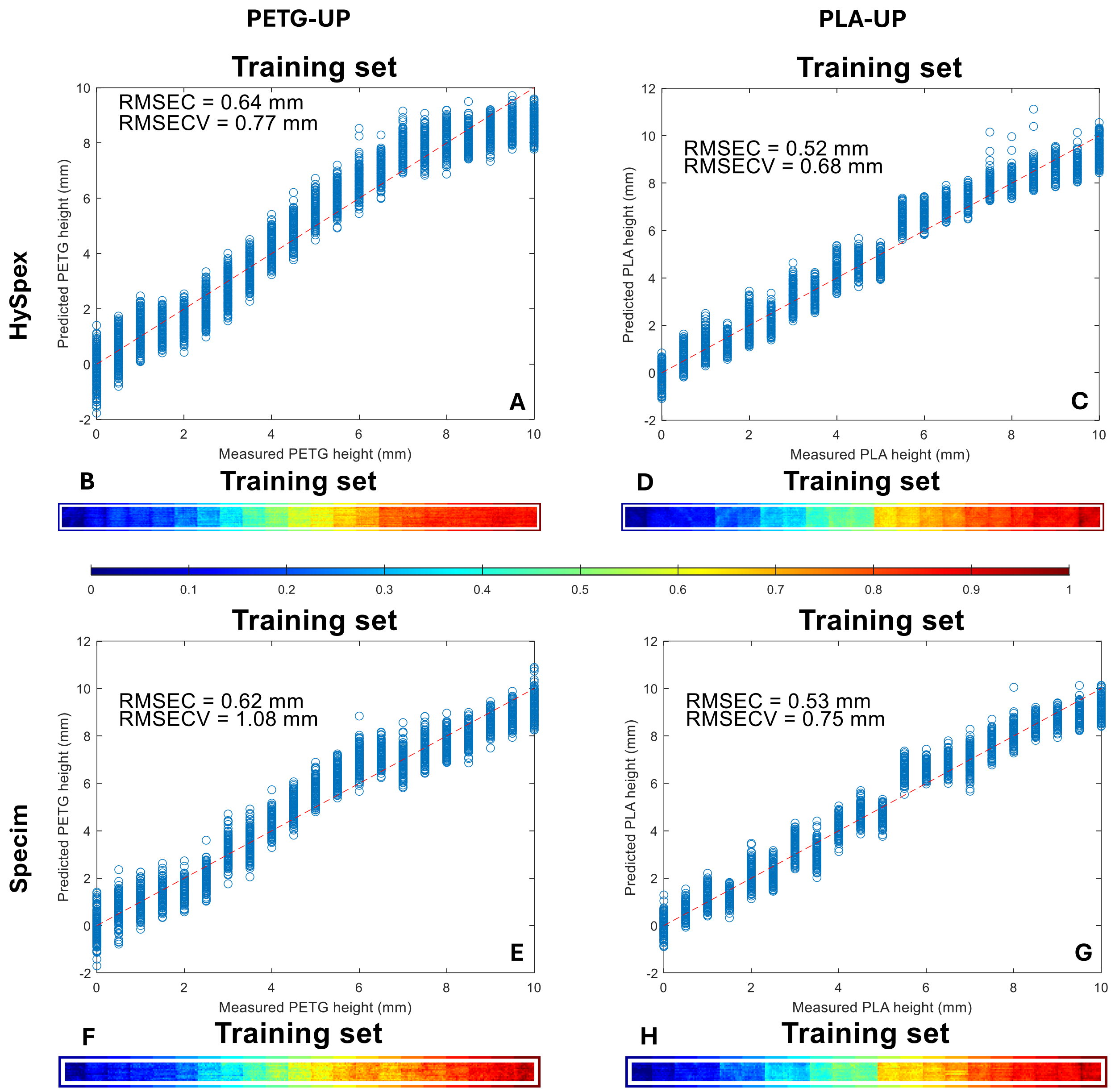

***Figure S4**: PLS regression results for HySpex (A-B-C-D) and Specim (E-F-G-H) data for PETG-UP (A-B-E-F) or PLA-UP (C-D-G-H) configuration. In particular, predicted versus measured polymer height plots (A, C, E, G) and prediction maps (B, D, F, H) for the training set are displayed. Each prediction map is displayed with a border which represents the colour the corresponding ROI should have if its prediction error was null.*

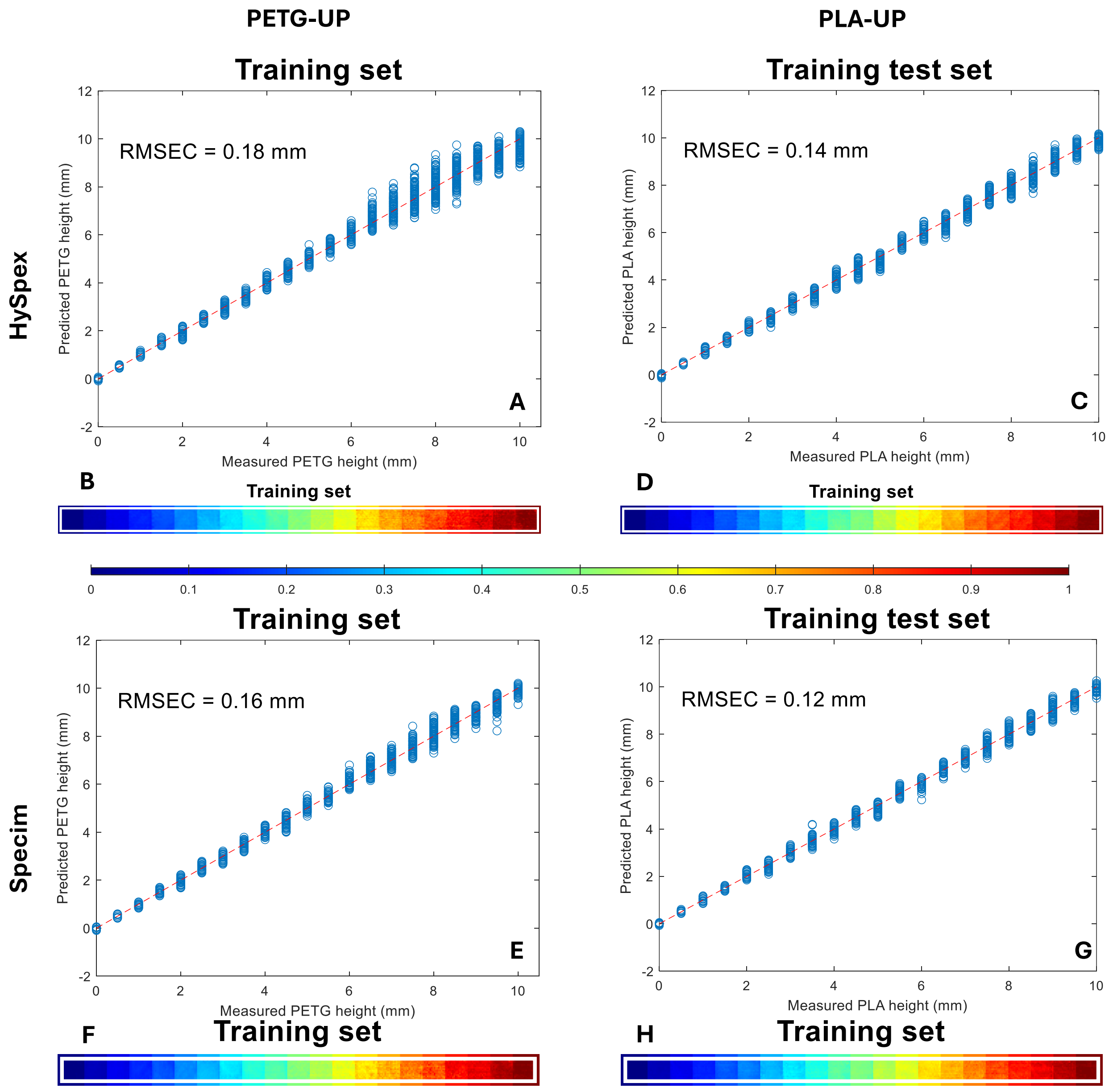

Figure S5: *CNN regression results for HySpex (A-B-C-D) and Specim (E-F-G-H) data for PETG-UP (A-B-E-F) or PLA-UP (C-D-G-H) configuration. In particular, predicted versus measured plots (A, C, E, G) and prediction maps (AB, D, F, H) for the training set are displayed. Each prediction map is reported with a border which represents the colour the corresponding ROI should have if its prediction error was null.*
